# Effects of Melatonin on Adrenal Cortical Functions of Indian Goats under Thermal Stress

**DOI:** 10.4061/2010/348919

**Published:** 2009-12-06

**Authors:** Veerasamy Sejian, Rajendra Swaroop Srivastava

**Affiliations:** ^1^Neurophysiology Laboratory, Division of Physiology and Climatology, Indian Veterinary Research Institute, Izatnagar, Bareilly 243122, India; ^2^Scientist, Adaptation Physiology Laboratory, Division of Physiology & Biochemistry, Central Sheep & Wool Research Institute, Avikanagar, Via-Jaipur, Rajasthan 304501, India

## Abstract

The study was conducted with the primary objective to establish the influence of melatonin on adrenocortical functions to ameliorate thermal stress in goats. Endocrine secretions and several other blood biochemical parameters reflecting the animals adrenocortical stress response were determined over a one-week period after goats had been exposed to 40°C and 60%RH for 10 days. The study was conducted for a period of 17 days in psychrometric chamber. The animals served as self-controls prior to start of the experiment. Blood samples were drawn on day 10 to establish effect of thermal stress. Chemical adrenalectomy was achieved using metyrapone followed by exogenous melatonin treatment. 40°C of thermal stress which is quite normal in tropical zone significantly (*P* ≤ .05) influenced all parameters except plasma insulin. Metyrapone treatment significantly (*P* ≤ .05) affected plasma levels of glucose, total protein, total cholesterol, cortisol, and aldosterone. Metyrapone aggravated thermal stress by decreasing cortisol level in goats. Melatonin treatment at 11:00 AM significantly (*P* ≤ .05) influenced plasma levels of glucose, total protein, total cholesterol, cortisol, aldosterone and insulin. Metyrapone treatment aggravated thermal stress although administration of melatonin could ameliorate the condition. This establishes the role of melatonin in relieving thermal stress in goats.

## 1. Introduction

The responses of animals to changes in environmental temperature emphasize the key difference between ruminant and nonruminant species in their comfort zones. Ruminants have wide comfort zones and a high degree of thermal tolerance so it is likely that climate change resulting in an increase of a few degrees is not going to have any major effect on these animal performances [1]. The exceptions would be at the extremes. Despite having well developed mechanisms of thermoregulation, ruminants do not maintain strict homeothermy under heat stress [2]. There is unequivocal evidence that hyperthermia is deleterious to any form of productivity, regardless of breed, and stage of adaptation. Although goats are known to be adapted to harsh environments [3] their productivity is affected adversely by extreme climatic conditions. Further it has been reported that goats with production demands are susceptible to heat stress in spite of heat resistant characteristics [4]. Previous studies on thermoregulatory response of goats indicated that the body temperature and respiration rate of goats increase with rise in ambient temperature [5]. Marked seasonal changes in rectal temperature, respiration rate, and energy expenditure were also reported in goats under tropical conditions [6]. Further there is evidence for deleterious effects on goat productivity when goats had been exposed over temperatures of 34–36°C [7]. 

 Pineal gland is considered as a neuroendocrine transducer of cyclic photic input, which is responsible for the seasonal changes in reproductive capability of various species [8]. Considerable evidence has now been accumulated to indicate its participation in a wide range of extra reproductive processes [9], among which Pineal-Adrenal, Pineal-Thyroid, and Pineal-Immune system, relationships are the thrust areas of research investigations. 

 The study of Pineal-Adrenal relationship probably began with Farrel's [10] discovery of adrenoglomerulotropin in the pineal extracts of rats. Since then, many investigators have attempted to establish this relationship [11–13]. Nevertheless, the contradictory nature of the results obtained so far makes it difficult to draw any definitive conclusions about the pineal-adrenal axis [14–16]. 

 The early suggestion of a functional antagonism between the pineal and the adrenal or between melatonin and corticosteroids became additionally reinforced by experimental and clinical findings indicating that melatonin may be able to protect the organism against stress induced pathologies such as gastric ulceration [17], involution of the thymus [18], and age-associated stress induced diseases [19]. 

 There is evidence that adrenalectomy in rats leads to significant increase in number of pinealocyte processes and an increase in melatonin forming enzyme HIOMT which ultimately leads to an increase in pineal endocrine activity [20]. Compensatory adrenal hypertrophy has been shown to be blocked by melatonin [21]. 

 Considering that both pineal and adrenal glands influences each other's function through their endocrine secretions [12, 13], the present study was conducted to establish the relationship between pineal gland and adrenal cortex under thermal stress. The primary objective of the study was to observe the modulating role of melatonin on adreno cortical functions to relieve thermal stress in goats.

## 2. Materials and Methods

### 2.1. Site of Study

The present study was conducted at the Division of Physiology and Climatology, IVRI. The institute is located at an altitude of 564 feet above mean sea level, at the latitude of 28° north and 79° east. The climate in this area is tropical monsoon, with peak summer between March to May and peak winter between November to January. The peak rainfall occurs between July to August. The experiment was carried out during March to May. The mean environmental temperature, relative humidity, and vapour pressure during the study period ranged between 37–45°C, 45–72 percent, and 7–15 mm Hg, respectively.

### 2.2. Animals

Six healthy female Marwari goats age 8–12 months and weighing 12–15 kgs were used in the present study. These animals served as self-controls prior to start of experiment. The animals were housed in well-ventilated sheds and maintained under proper hygienic conditions. The animals had ad libitum access to feed and good quality drinking water.

### 2.3. Experimental Design

The study was conducted for a period of 17 days. First 10 days were used to induce thermal stress. Between Day 11 to Day 17 drug administration was carried out. Goats had been exposed to 40°C and 60% relative humidity in the psychrometric chamber for four hours a day from 9:00 h to 13:00 h on each day of the 17-day trial period. Prior to start of experiment, blood samples were drawn from all six animals on Day 0 at 11:00 h to establish the control values for the parameters studied. Blood samples were collected after thermal exposure on 10th day at 11:00 h to establish heat stress effect on various parameters studied. On the 11th day Metyrapone (2-Methyl-1, 2-di-3-pyridyl-1-propanone; Aldrich, USA) was administered intravenously in these animals at the dose rate of 100-mg/kg BW to induce chemical adrenalectomy. Metyrapone blocks 11*β* hydroxylase, which catalyzes the conversion of 11- deoxycortisol to cortisol, the final step in cortisol biosynthesis. Metyrapone injection was given at 10:00 h. After one hour of metyrapone treatment at 11:00 h, five mL blood was drawn from jugular vein to ascertain chemical adrenalectomy. Melatonin (Sigma, USA) was administered intravenously at the dose rate of 0.1 mg/Kg BW to counteract the metyrapone effect. The doses of both metyrapone and melatonin were established as per our previous study in our laboratory [22]. Time of melatonin injection was at 11:00 h. After an hour of melatonin administration, five mL blood was drawn from jugular vein to observe the stress relieving capability of melatonin. This procedure was followed from 11th to 17th days of the study. Samples were drawn after one hour of each drug administration to ensure complete action of these drugs to induce changes in parameters studied as the half-life of both metyrapone and melatonin was between 30 minutes to one hour. Plasma was separated from blood by centrifugation at 4500 rpm at room temperature for 20 minutes. The plasma was then divided into aliquots in microcentrifuge tubes, and kept frozen at −20°C till further analysis. The study was conducted after obtaining due approval from the institutes ethical committee for subjecting the animal to thermal stress and metyrapone treatment. The present study is the one in a series of experiment conducted to establish pineal influence on other endocrine gland secretions under thermal stress. The animals used in the present study were the same that were used in the previous studies. The present study is an extension from the previous one, focusing in adrenal and metabolic responses after exogenous melatonin treatment in goats under thermal stress.

### 2.4. Parameters Studied

The parameters analyzed in the study were plasma glucose, total protein, total cholesterol, urea, melatonin, cortisol, corticosterone, aldosterone, and insulin. Plasma glucose [23], total plasma protein [24], total plasma cholesterol [25], and plasma urea [26] were estimated using *Span* diagnostic kits, India as per standard method using the Semi-auto analyzer (ERBA *CHEM*-5 Plus). All the biochemical parameters included in the study were controlled by adrenal glucocorticoids. Glucocorticoids favours hepatic gluconeogenesis and protein catabolism to maintain homeostasis. Hence glucocorticoids have direct control over glucose and protein metabolism and hence these parameters were included in addition to urea in the present study. Glucocorticoids are steroid hormones and since cholesterol is the precursor material for steroid hormone production, it was included in the study as glucocorticoid levels were greatly influenced under thermal stress. Insulin has direct control over glucose level in the body and hence it was included in the study. Cortisol and aldosterone are endocrine parameters of adrenal cortex and hence these parameters are obvious for the study. These parameters are included in the study to know the influence of pineal hormone melatonin on these parameters and further to know whether exogenous melatonin modulates the adrenal cortex activity to influence the levels of these parameters to ensure thermoregulation under exposure to constant heat stress. 

 Hormonal parameters such as cortisol (analytical sensitivity was 10 nM; the intra-assay and inter-assay coefficients of variations were 5.8% and 9.2%, resp.), aldosterone (analytical sensitivity was 6 pg/mL; the intra-assay and inter-assay coefficient of variations were 9.5% and 9.9%, resp.), and insulin (analytical sensitivity was 0.5 *μ*IU/mL; the intra-assay and inter-assay coefficients of variations were 4.3% and 3.4%, resp.), were estimated by RIA using the Packard Cobra II gamma counter employing RIA kits supplied by Immunotech, France. Cortisol RIA assay was validated for parallelism and recovery for goats in our laboratory as described by Vakkuri et al. [27]. Aldosterone and insulin RIA assays were validated as per methodology of Sejian et al. [28].

### 2.5. Data Analysis

The data obtained was analyzed statistically by analysis of variance for repeated measures using SPSS -15 software. Significant differences were determined at the levels of *P* ≤ .05.

## 3. Results

Chemical adrenalectomy was induced using metyrapone and sufficient time was given to observe its effects. Then exogenous melatonin was administered to relieve the metyrapone effects in these animals. The results obtained are being discussed elaborately for each parameter as follows. 

 The mean plasma glucose level increased significantly (*P* ≤ .05) after thermal exposure (Table 1). Metyrapone treatment decreased significantly (*P* ≤ .05) the mean plasma glucose (Table 2). But melatonin treatment was able to increase the plasma glucose significantly (*P* ≤ .05) when compared to thermal stress value. This trend continued until the end of experimental period. The mean total plasma protein significantly (*P* ≤ .05) decreased after thermal exposure (Table 1). On day 11, metyrapone treatment increased significantly (*P* ≤ .05) while melatonin treatment reduced significantly (*P* ≤ .05) the level of total protein (Table 2). This trend continued up to 17th day. Mean total plasma cholesterol increased significantly (*P* ≤ .05) after thermal exposure (Table 1). Metyrapone treatment significantly (*P* ≤ .05) increased total plasma cholesterol throughout the study period while melatonin treatment decreased cholesterol values (Table 2). However this reduction in cholesterol level brought about by melatonin is not statistically significant. Mean plasma urea increased significantly (*P* ≤ .05) after thermal exposure (Table 1). Metyrapone treatments increased plasma urea while melatonin treatment decreased urea values throughout the study period (Table 2). This effect of both metyrapone and melatonin on urea level was significant (*P* ≤ .05) on majority of experimental days. 

 Mean plasma cortisol significantly (*P* ≤ .05) increased after thermal exposure (Table 1). Metyrapone reduced significantly (*P* ≤ .05) the level of cortisol while melatonin further reduced significantly (*P* ≤ .05) the level of cortisol (Table 3). This trend remained on all days of the experiment. Mean plasma aldosterone showed significant (*P* ≤ .05) reduction after thermal exposure (Table 1). Metyrapone decreased significantly (*P* ≤ .05) while melatonin further decreased significantly (*P* ≤ .05) the level of aldosterone (Table 3). On the last day metyrapone treatment significantly (*P* ≤ .05) increased the aldosterone level to 5.40 ± 1.43 pg/mL while melatonin reduced this increase significantly (*P* ≤ .05) to 3.52 ± 0.99 pg/mL and brought the value towards the control (Table 3). Mean plasma insulin decreased significantly (*P* ≤ .05) after thermal exposure (Table 1). Both metyrapone and melatonin treatment increased plasma insulin (Table 3). These effects of metyrapone and melatonin were significant on days 12 and 16 while on rest of the experimental days the effects were nonsignificant. 

## 4. Discussion

Melatonin could reverse the combined effects of both thermal stress as well as metyrapone treatment on plasma glucose. Melatonin increased the metyrapone induced reduction in plasma glucose. Our finding coincided with the findings of Poon et al. [29], Fabis et al. [30], Markova et al. [31], and Bojkova et al. [32] who established significant increase in plasma glucose after melatonin treatment in rats. With respect to farm animals, our finding is similar to that obtained by Darul and Kruczynska [33, 34] in goat and cow. They found significant increase in plasma glucose after exogenous melatonin treatment in goat and cow. Poon et al. [29] explained the possible mechanism by which melatonin controlled the glucose level. They explained a possible direct action of melatonin on hepatocytes to modulate the plasma glucose. Their hypothesis was supported by the presence of high affinity melatonin receptors in the mouse hepatocytes. Total plasma protein showed significant (*P* ≤ .05) reduction for metyrapone treatment. The reason for this could be deficiency of glucorticoids caused by metyrapone. Glucocorticoids favour protein catabolism. Its deficiency leads to increase in total plasma protein. Our finding coincides with the findings of Shivkumar et al. [35]. They established significant increase in total plasma protein after unilateral adrenalectomy in goats. But melatonin treatment was able to successfully reverse the effect caused by metyrapone with respect to total plasma protein level. Total plasma cholesterol showed highly significant changes to both metyrapone and melatonin treatments. Metyrapone treatment increased the cholesterol level. This finding coincided with the findings of Shivkumar et al. [35]. They reported that after unilateral adrenalectomy there was an increase in total cholesterol level. Since cholesterol was the precursor of steroid synthesis, reduction in glucocorticoid synthesis due to metyrapone treatment leads to unutilization of cholesterol. But melatonin treatment significantly (*P* ≤ .05) reduced this increase in cholesterol caused by metyrapone treatment. Although the melatonin reduces the cholesterol level from that caused by metyrapone, it is still above the control level until the end of the experiment. Darul and Kruczynska [33, 34] also reported high increase in plasma cholesterol after exogenous melatonin treatment in goat and cow. The increase in plasma urea after metyrapone treatment is very similar to the results reported by Shivkumar et al. [35] in goats. However melatonin treatment reduced this increase in urea level caused by metyrapone treatment. 

 Metyrapone significantly reduced the cortisol level, with the principal hormone having antistress effects. This could have lead to aggravation of thermal stress due to reduced cortisol concentration to relieve thermal stress. Exogenous melatonin further reduced cortisol level. This trend remained for the entire study period. Our report contradicted the findings of Cagnacci et al. [36], that melatonin treatment lead to increase in daytime cortisol in humans. This difference could be due to the extreme heat stress effects in our study. Torres-Farfan et al. [37] reported mt1 melatonin receptor in the adrenal cortex of capuchin monkey and further they found out that melatonin by acting on this receptor inhibits ACTH stimulated cortisol production. These evidences suggest that melatonin action in controlling cortisol production in the present study could be receptor mediated. Further Konakchieva et al. [38] indicated that melatonin attenuates the adrenocortical response to stress and influences the biosynthesis, release and glucocorticoid responsiveness to hypothalamic ACTH secretion. 

 Both metyrapone and melatonin treatments showed highly significant changes in aldosterone level. In a peculiar manner both the treatments was able to increase aldosterone on the last day, and melatonin treatment brought the aldosterone level towards control. Melatonin treatment significantly increased the insulin level. Darul and Kruczynska [33, 34] reported similar finding in cows after melatonin administration. The effect of melatonin on insulin level in the present study could be due to direct action of melatonin on pancreas. This view was strongly supported by the fact that pancreas posses the receptor for melatonin action as reported by Peschke et al. [39]. 

 Exogenous melatonin administered had a protective role on the parameters studied. There are reports which suggest heat stress relieving effects of melatonin when administered exogenously during day time [40, 41]. Our results from the experiment also points towards the existence of such heat stress relieving role of melatonin. The results obtained reveal the chemical adrenalectomy relieving effects of the melatonin. This shows that during thermal stress melatonin were able to successfully modify the adrenal cortex functions to relieve thermal stress. Given the importance of thermal stress in hampering animal productivity to a greater extent in tropical countries, our finding has greater significance in terms of improving economy of farm households as well as poor farmers are concerned. The data generated from this study help us to understand the functional relationship between pineal and adrenal glands, and how they influence each other for the well being of the domestic and farm animals. Further detailed studies are required though to understand the mechanisms of interactions involved between these glands.

## Figures and Tables

**Table 1:** Mean and SE of biochemical and endocrine parameters studied in control and heat exposed goats. Mean and SE are based on 6 animals.

Parameters	Control (day 0)	Heat stress (day 10)
Glucose (mg/dL)	36.47 ± 2.91	51.56 ± 3.64^(A)^
Total protein (g/dL)	7.31 ± 0.30	6.89 ± 0.21^(A)^
Total cholesterol (mg/dL)	143.26 ± 3.13	157.79 ± 3.94^(A)^
Urea (mg/dL)	65.77 ± 4.31	73.44 ± 3.13^(A)^
Cortisol (nmol/L)	18.76 ± 2.44	82.74 ± 4.33^(A)^
Aldosterone (Pg/mL)	3.60 ± 0.89	1.46 ± 0.27^(A)^
Insulin (micro IU/mL)	9.16 ± 0.75	7.22 ± 1.12

^(A)^Indicates statistical significance at *P* ≤ .05 when compared with control (day 0) Value.

**Table 2:** Mean and SE of biochemical parameters in blood plasma of heat exposed, chemically adrenalectomized, and melatonin administered goats. Values are mean and SEM.

Experiment days	Treatment	Glucose (mg/dL)	Total protein (g/dL)	Total cholesterol (mg/dL)	Urea (mg/dL)
Heat stress (day 10)	—	51.56 ± 3.64	6.89 ± 0.21	157.79 ± 3.94	73.44 ± 3.13
Day 11	MET^(1)^	35.58 ± 7.69^(A)^	8.77 ± 0.30^(A)^	174.18 ± 3.03^(A)^	84.33 ± 3.45^(A)^
	MEL^(2)^	43.73 ± 3.04	7.42 ± 0.24^(A)^*	151.63 ± 2.62*	66.82 ± 1.80^(A)^*
Day 12	MET	39.85 ± 4.98^(A)^	8.02 ± 0.06^(A)^	189.45 ± 3.49^(A)^	65.92 ± 4.59^(A)^
	MEL	40.19 ± 1.32^(A)^	7.67 ± 0.16^(A)^*	161.18 ± 2.63*	55.41 ± 1.82^(A)^*
Day 13	MET	47.65 ± 3.85	7.85 ± 0.26^(A)^	187.61 ± 7.21^(A)^	76.66 ± 2.84
	MEL	49.70 ± 1.38	7.31 ± 0.30^(A)^*	165.63 ± 4.96*	69.88 ± 5.25
Day 14	MET	48.73 ± 5.33	7.78 ± 0.15^(A)^	188.74 ± 6.04^(A)^	74.47 ± 4.70
	MEL	69.33 ± 2.81^(A)^*	7.52 ± 0.22^(A)^	157.73 ± 3.46*	63.02 ± 4.84^(A)^*
Day 15	MET	35.38 ± 2.82^(A)^	8.24 ± 0.24^(A)^	159.62 ± 3.61	67.96 ± 5.92
	MEL	64.30 ± 2.00^(A)^*	7.62 ± 0.19^(A)^*	149.97 ± 3.86	62.56 ± 5.37^(A)^
Day 16	MET	38.38 ± 4.66^(A)^	7.98 ± 0.26^(A)^	178.15 ± 3.57^(A)^	78.81 ± 4.58
	MEL	47.65 ± 2.84*	7.38 ± 0.27^(A)^*	162.42 ± 4.86*	70.41 ± 3.30*
Day 17	MET	35.47 ± 3.11^(A)^	8.45 ± 0.28^(A)^	178.43 ± 2.60^(A)^	74.51 ± 4.37
	MEL	44.67 ± 2.61*	7.50 ± 0.17^(A)^*	159.64 ± 2.98*	70.84 ± 2.38

^(1)^Metyrapone; ^(2)^Melatonin. ^(A)^Indicates statistical significance at *P* < .05 when compared with day 10 value. *Indicates statistical significance at *P* < .05 when compared with corresponding MET value on the same day.

**Table 3:** Mean and SE of cortisol, aldosterone, and insulin in blood plasma of heat exposed, chemically adrenalectomized, and melatonin administered goats. Values are mean and SEM.

Experiment days	Treatment	Cortisol (nmol/L)	Aldosterone (Pg/mL)	Insulin (micro IU/mL)
Heat stress (day 10)	—	82.74 ± 4.33	1.46 ± 0.27	7.22 ± 1.12
Day 11	MET^(1)^	61.94 ± 4.94^(A)^	0.31 ± 0.11^(A)^	9.11 ± 1.53
	MEL^(2)^	48.87 ± 4.93^(A)^*	0.18 ± 0.06^(A)^	19.43 ± 7.74^(A)^
Day 12	MET	67.94 ± 3.82^(A)^	0.29 ± 0.05^(A)^	18.43 ± 1.16^(A)^
	MEL	62.97 ± 4.11^(A)^	0.28 ± 0.10^(A)^	26.43 ± 1.23^(A)^
Day 13	MET	79.23 ± 4.45	0.70 ± 0.19^(A)^	13.77 ± 3.19
	MEL	72.88 ± 3.84^(A)^	0.19 ± 0.05^(A)^	31.50 ± 7.22^(A)^*
Day 14	MET	82.80 ± 4.90	0.54 ± 0.20^(A)^	10.82 ± 1.57
	MEL	75.93 ± 5.84	1.04 ± 0.70	29.82 ± 13.10^(A)^*
Day 15	MET	86.86 ± 6.35	0.34 ± 0.09^(A)^	8.25 ± 1.11
	MEL	68.18 ± 4.21^(A)^*	0.26 ± 0.10^(A)^	18.77 ± 0.40^(A)^
Day 16	MET	72.68 ± 5.52^(A)^	0.62 ± 0.23^(A)^	18.50 ± 2.84^(A)^
	MEL	56.42 ± 3.65^(A)^*	0.29 ± 0.12^(A)^	23.54 ± 4.49^(A)^
Day 17	MET	84.89 ± 8.01	5.40 ± 1.43^(A)^	13.62 ± 3.66
	MEL	55.64 ± 3.79^(A)^*	3.52 ± 0.99^(A)^*	14.31 ± 2.45

^(1)^Metyrapone; ^(2)^Melatonin. ^(A)^Indicates statistical significance at *P* < .05 when compared with day 10 value. *Indicates statistical significance at *P* < .05 when compared with corresponding MET value on the same day.
